# Supplementary material for: Cholecystokinin Receptor Antagonist Induces Pancreatic Stellate Cell Plasticity Rendering the Tumor Microenvironment Less Oncogenic
Source: Cancers (Basel). 2023 May 18;15(10):2811. doi: 10.3390/cancers15102811 (PMC10216345; doi:10.3390/cancers15102811)
Supplement: Supplementary file 1 [file cancers-15-02811-s001.zip › cancers-2307362-supplementary.pdf]

Supplementary Data

Supplementary Methods: Measurement of proline and 4-hydroxyproline by mass spectroscopy

MS parameters

An MRM based mass spectrometry method was developed for the measurement of proline and 4-hydroxyproline using multiple reactions monitoring mass spectrometry (MRM-MS) in conjunction with ultra-performance liquid chromatography (UPLC). The column eluent was introduced directly into the TQS mass spectrometer by electrospray ionization (ESI+) at a capillary voltage of 2.97 kV and a sampling cone voltage of 101 V. The desolvation gas flow was set to 1000 L/h and the desolvation temperature was set to 500 °C. The cone gas flow was 150 L/h and the source temperature was set to 150 °C. The cone voltage and collision energies were optimized for the analyte to obtain maximum ion intensity for parent and daughter ions using "IntelliStart" feature of MassLynx software (Waters Corporation). The instrument parameters were optimized to gain maximum specificity and sensitivity of ionization for the parent and daughter ions. Signal intensities from the MRM Q1>Q3 ion pairs for the drug proline (116.1>69.9, 4- hydroxyproline (132.1>67.9), and proline-2,5,5-d3 (119>70) were ranked to ensure selection of the most intense precursor and fragment ion pair for MRM-based quantitation. Figure S1 shows the representative chromatogram for proline, 4-hydroxyproline and the internal standard (proline-2,5,5-d3).

Data acquisition details

Prior to data acquisition, sample queue was randomized. A 7 point calibration curve was used to extrapolate relative ,metabolite concentrations in experimental Figure S2 (details in Table S1) and Figure S3 (details in Table S2) shows the calibration curves for proline and 4-hydroxyproline, respectively. The linearity of drug response in calibration curve points was ascertained by including three quality control (QC) samples at the start and in the end of the batch. Standard QC and pooled QC samples were injected periodically to monitor the consistency in the drug response for a particular drug concentration and monitor instrumental variance. Blanks (solvent alone) were injected on either side of test samples to assess sample-to-sample metabolite carryover. As a measure of assay reproducibility and drug stability and to study inter day variations, the data was acquired for two consecutive days. Data were processed using Target Lynx 4.1. The relative quantification values of analytes were determined by calculating the ratio of peak areas of transitions of samples normalized to the peak area of the internal standard.

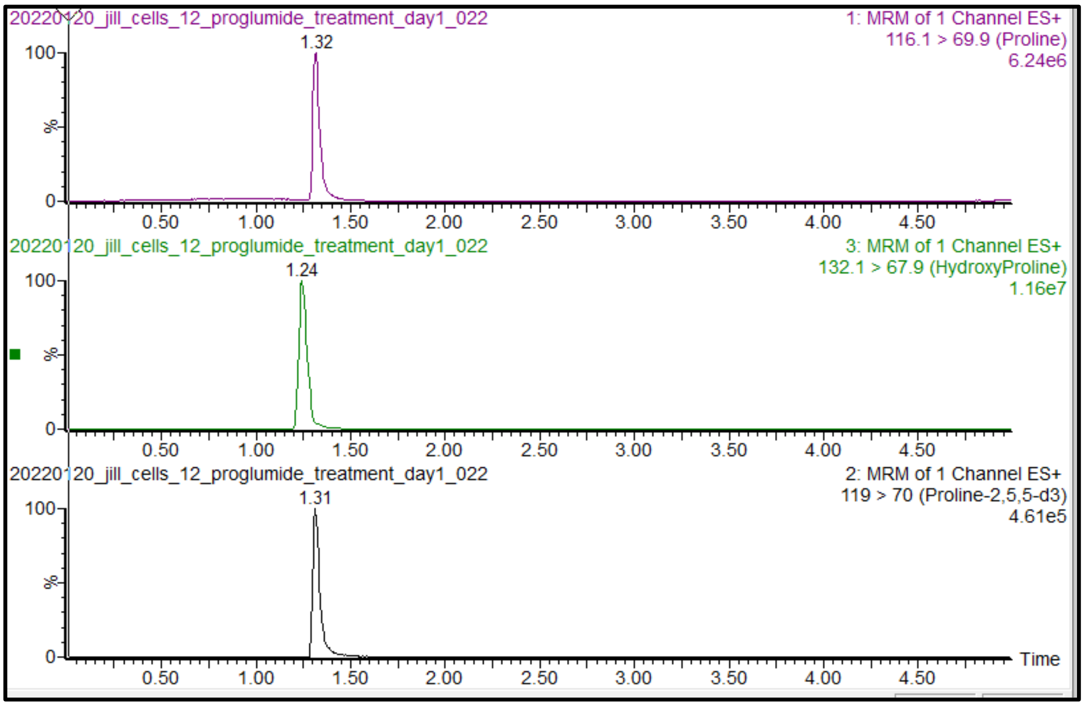

Supplementary Figure S1. UPLC-MRM based mass spectrometric analysis: A sample chromatogram of eluted proline, 4-hydroxyproline and internal standard (proline-2,5,5-d3).

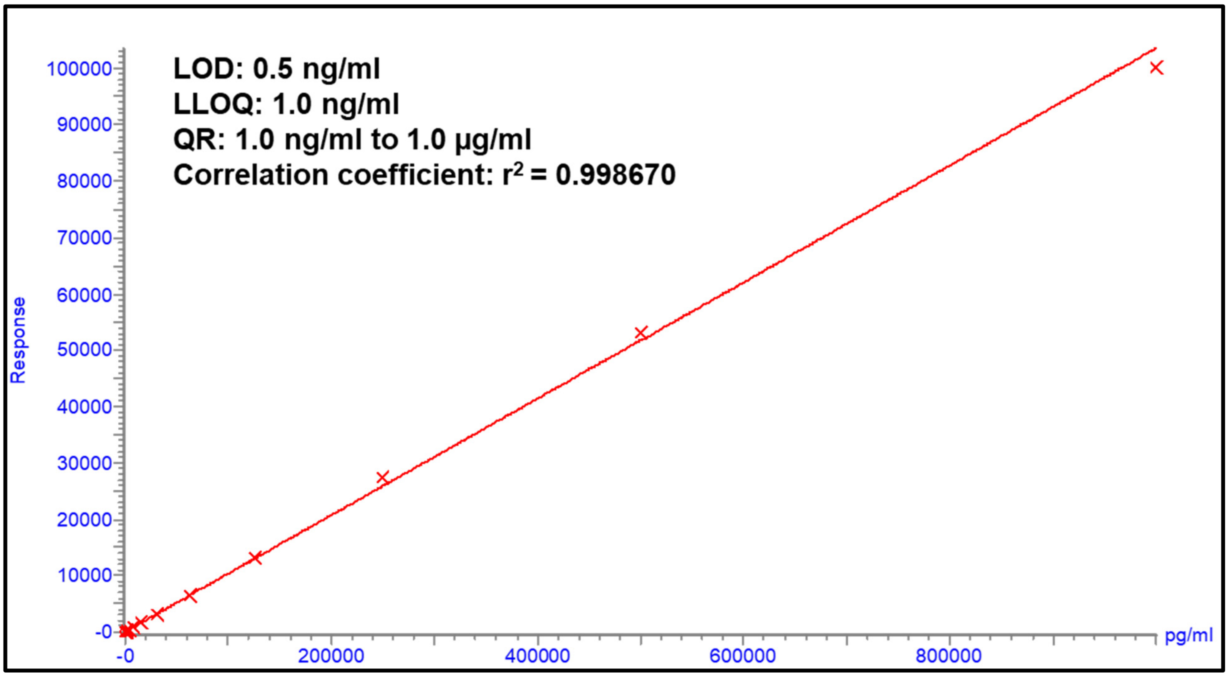

Supplementary Figure S2. UPLC-MRM mass spectrometry analysis of proline: Calibration curve for proline over a range from 1.0 ug/ml to 1.0 ng/ml shows linearity. (LOD= limit of detection; LLOQ= lower limit quantification, QR; Quantification range).

Supplementary Table S1. UPLC-MRM based mass spectrometric analysis of proline in mPSCs over a range from 0.5 ng/ml to 1.0 µg/ml: Calibration curve details

Std. Conc (pg/ml)	RT	Area	IS Area	Response	Conc (pg/ml)	%Dev
488	1.26	2732.6	19958.1	136.9	1334.7	173.5
976	1.31	2125.9	19995.2	106.3	1039.2	6.5
1950	1.31	3604.3	20356.3	177.1	1722.5	-11.7
3900	1.31	8000.4	20104.6	397.9	3855.9	-1.1
7810	1.31	15384.0	20056.6	767.0	7420.8	-5
15600	1.31	32897.9	19718.7	1668.4	16126.6	3.4
31250	1.32	63942.5	19906.0	3212.2	31038.3	-0.7
62500	1.32	132809.0	20325.5	6534.1	63123.4	1
125000	1.32	268593.5	20416.0	13156.1	127083.2	1.7
250000	1.31	533200.4	19374.1	27521.3	265833.2	6.3
500000	1.32	1066118.5	20022.5	53246.1	514302.8	2.9
1000000	1.32	2070331.1	20670.1	100160.9	967440.0	-3.3

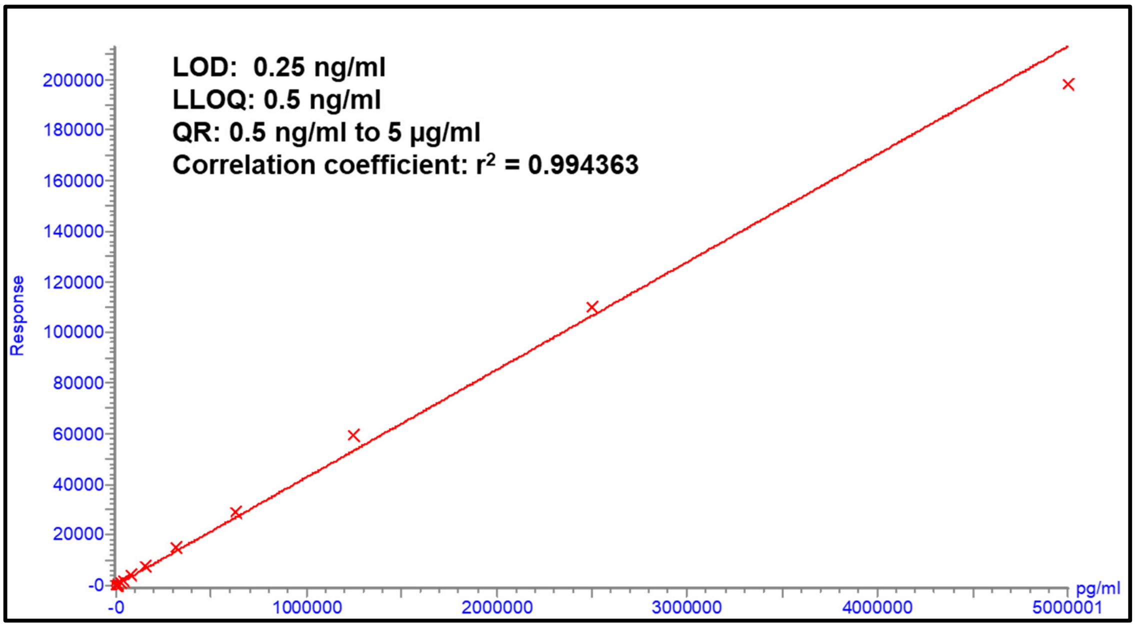

Supplementary Figure S3. UPLC-MRM mass spectrometry analysis of 4-hydroxyproline: Calibration curve for 4-hydroxyproline over a range from 5.0 ug/ml to 0.5 ng/ml shows linearity. (LOD= limit of detection; LLOQ= lower limit quantification, QR; Quantification range).

Supplementary Table S2. UPLC-MRM based mass spectrometric analysis of 4-hydroxyproline in mPSCs over a range from 0.3 ng/ml to 5.0 µg/ml: Calibration curve details

Std. Conc (pg/ml)	RT	Area	IS Area	Response	Conc (pg/ml)	%Dev
**305.0**	1.2	423.5	20156.1	21.0	166.3	-45.5
**610.0**	1.3	718.4	20008.4	35.9	515.9	-15.4
**1220.0**	1.3	1294.8	20136.7	64.3	1182.6	-3.1
**2440.0**	1.2	2256.5	19958.1	113.1	2327.5	-4.6
**4880.0**	1.2	4460.9	19995.2	223.1	4911.1	0.6
**9760.0**	1.2	8739.7	20356.3	429.3	9753.4	-0.1
**19500.0**	1.2	17948.7	20104.6	892.8	20634.3	5.8
**39050.0**	1.2	36944.4	20056.6	1842.0	42921.6	9.9
**78100.0**	1.3	74480.5	19718.7	3777.2	88357.0	13.1
**156200.0**	1.3	148517.1	19906.0	7460.9	174848.4	11.9
**312500.0**	1.3	299062.3	20325.5	14713.6	345135.7	10.4
**625000.0**	1.3	591398.3	20416.0	28967.5	679802.5	8.8
**1250000.0**	1.3	1152080.1	19374.1	59465.0	1395856.0	11.7
**2500000.0**	1.2	2202685.8	20022.5	110010.8	2582626.1	3.3
**5000000.0**	1.3	4094425.0	20670.1	198084.9	4650526.3	-7.0

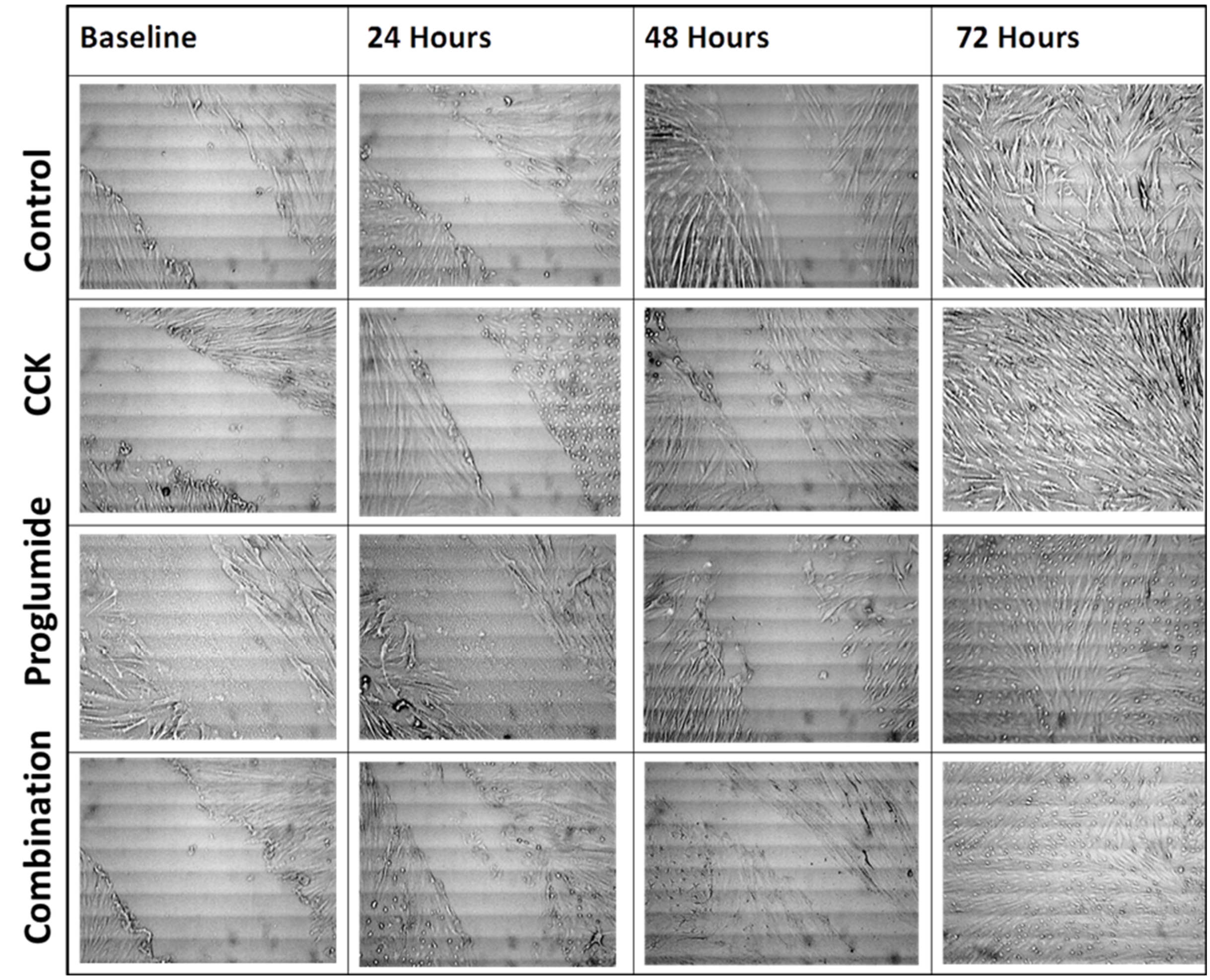

Supplementary Figure S4. Migration assay of human PSCs. Representative photos taken at baseline, 24 hrs, 48 hrs, and 72 hrs are shown for human PSC treated with media alone (control) CCK, proglumide, or the combination of CCK and proglumide. Images taken at 4X magnification with a Moticam 1sp: 1.3mp digital camera.

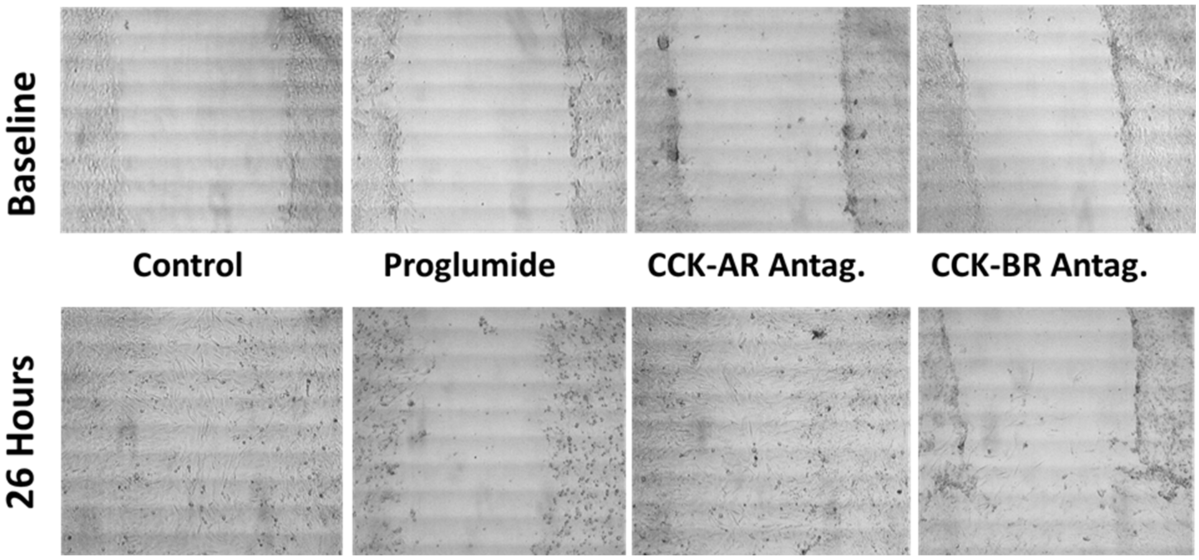

Supplementary Figure S5. Migration assay of mouse PSC treated with proglumide or selective CCK-AR or CCK-BR antagonist. Representative images obtained at baseline, (time 0 hours, and again after 26 hours after treatment of mPSCs with media alone (Control), proglumide, the CCK-AR antagonist (L264,718) or the CCK-BR antagonist (L365,260).

Supplementary Table S3. Relative mRNA expression of differentially expressed genes from untreated mPSCs (control) of mPSCs treated with CCK (10 nM), Proglumide (20 nM), or the combination of CCK and proglumide (Combo). Data normalized using *Hprt* (* $p < 0.05$).

Gene	Control (n=3)	CCK (n=3)	Prog (n=3)	Combo (n=3)
	Mean ± SEM	Mean ± SEM	Mean ± SEM	Mean ± SEM
Hic-5	0.79 ± 0.16	0.93 ± 0.08	0.54 ± 0.09	0.62 ± 0.09
		P=0.47	*P*=0.25	*P*=0.41
Fap	1.29 ± 0.15	0.98 ± 0.05	1.11 ± 0.12	1.0 ± 0.09
		P=0.12	*P*=0.41	*P*=0.24
IL-1β	1.29 ± 0.14	0.93 ± 0.31	0.79 ± 0.20	1.64 ± 0.63
		P=0.35	*P*=0.11	*P*=0.61
mCol4α	1.42 ± 0.29	0.38 ±0.017	0.43 ±0.082	2.30 ± 1.64
		**P*=0.022	**P*=0.029	*P*=0.62

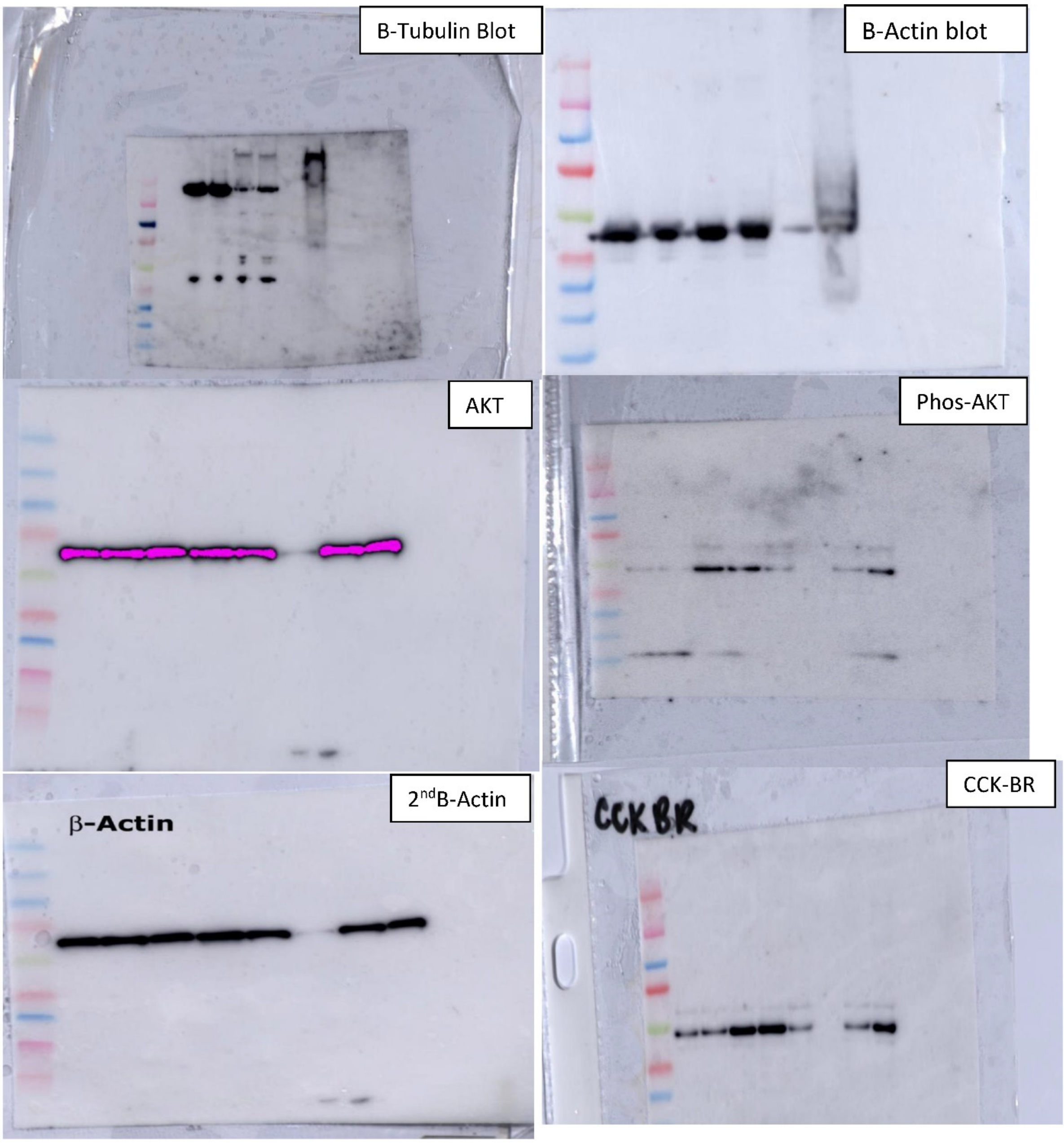
B-Tubulin Blot
B-Actin blot
AKT
Phos-AKT
2nd B-Actin
β-Actin
CCK-BR
CCKBR

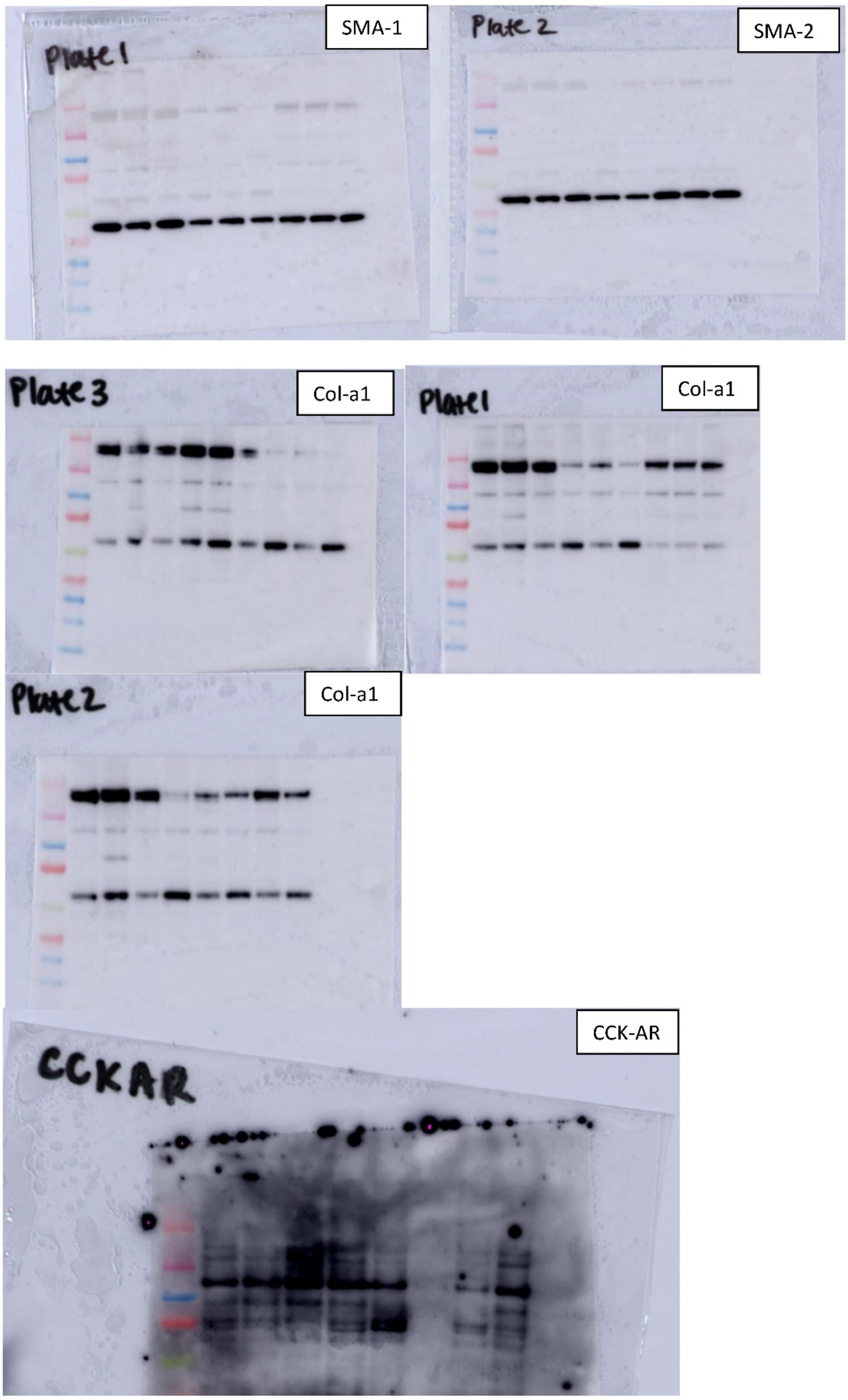

Supplementary Figure S6. The whole western blot.
